# Establishment of a Publicly Available Core Genome Multilocus Sequence Typing Scheme for Clostridium perfringens

**DOI:** 10.1128/Spectrum.00533-21

**Published:** 2021-10-27

**Authors:** Mostafa Y. Abdel-Glil, Prasad Thomas, Jörg Linde, Keith A. Jolley, Dag Harmsen, Lothar H. Wieler, Heinrich Neubauer, Christian Seyboldt

**Affiliations:** a Institute of Bacterial Infections and Zoonoses, Friedrich-Loeffler-Institut, Jena, Germany; b Department of Pathology, Faculty of Veterinary Medicine, Zagazig University, Zagazig, Sharkia Province, Egypt; c Department of Zoology, University of Oxford, Oxford, United Kingdom; d Department of Periodontology and Operative Dentistry, University Hospital Muenster, Muenster, Germany; e Robert Koch-Institut, Berlin, Germany; USDA-ARS

**Keywords:** *Clostridium perfringens*, SNP, cgMLST, genome typing

## Abstract

Clostridium perfringens is a spore-forming anaerobic pathogen responsible for a variety of histotoxic and intestinal infections in humans and animals. High-resolution genotyping aiming to identify bacteria at strain level has become increasingly important in modern microbiology to understand pathogen transmission pathways and to tackle infection sources. This study aimed at establishing a publicly available genome-wide multilocus sequence-typing (MLST) scheme for C. perfringens. A total of 1,431 highly conserved core genes (1.34 megabases; 50% of the reference genome genes) were indexed for a core genome-based MLST (cgMLST) scheme for C. perfringens. The scheme was applied to 282 ecologically and geographically diverse genomes, showing that the genotyping results of cgMLST were highly congruent with the core genome-based single-nucleotide-polymorphism typing in terms of resolution and tree topology. In addition, the cgMLST provided a greater discrimination than classical MLST methods for C. perfringens. The usability of the scheme for outbreak analysis was confirmed by reinvestigating published outbreaks of C. perfringens-associated infections in the United States and the United Kingdom. In summary, a publicly available scheme and an allele nomenclature database for genomic typing of C. perfringens have been established and can be used for broad-based and standardized epidemiological studies.

**IMPORTANCE** Global epidemiological surveillance of bacterial pathogens is enhanced by the availability of standard tools and sharing of typing data. The use of whole-genome sequencing has opened the possibility for high-resolution characterization of bacterial strains down to the clonal and subclonal levels. Core genome multilocus sequence typing is a robust system that uses highly conserved core genes for deep genotyping. The method has been successfully and widely used to describe the epidemiology of various bacterial species. Nevertheless, a cgMLST typing scheme for Clostridium perfringens is currently not publicly available. In this study, we (i) developed a cgMLST typing scheme for C. perfringens, (ii) evaluated the performance of the scheme on different sets of C. perfringens genomes from different hosts and geographic regions as well as from different outbreak situations, and, finally, (iii) made this scheme publicly available supported by an allele nomenclature database for global and standard genomic typing.

## INTRODUCTION

Clostridium perfringens is a Gram-positive anaerobic bacterium that is widely distributed in the soil and feces of humans and animals (1). C. perfringens produces resistant spores that allow the bacterium to survive in harsh environments and play a central role in the epidemiology of C. perfringens diseases (2). This bacterium produces a wide array of extracellular toxins and enzymes. Based on the presence of six typing toxins {α, β, ε, ι, *C. perfringens* enterotoxin [CPE] and necrotic enteritis B-like toxin [NetB]}, C. perfringens is classified into seven toxinotypes (A to G) (3). The toxins used for typing are plasmid encoded except α-toxin, which is chromosomally encoded, and CPE, where the gene can be located on a chromosome or a plasmid (4). Of the seven toxinotypes, C. perfringens type A is the most common and is widely isolated from healthy individuals and the environment. The diseases caused by type A strains are diverse, including traumatic gas gangrene in several mammalian species for which α-toxin and θ-toxin are important in disease progression (5). Type A also causes a variety of enteric infections in domestic animals, e.g., yellow lamb disease in sheep and necrohemorrhagic enteritis in calves (1, 6). In contrast to type A, toxinotypes B to G are associated with the incidence of certain diseases in specific host(s). For example, C. perfringens type B strains cause fatal hemorrhagic dysentery in lambs (1, 4), while type C strains cause necrotic enteritis and enterotoxaemia in lambs, piglets, calves, and foals. Type C strains that produce CPE and β-toxin are associated with foodborne illness in humans known as Pigbel or Darmbrand. C. perfringens type D is implicated in enterotoxaemia in sheep and goats (4). Type E strains are occasionally associated with calf enterotoxaemia and hemorrhagic enteritis (4). Type F strains produce CPE that is particularly important in C. perfringens food poisoning as well as nonfoodborne gastroenteritis in humans. C. perfringens type G describes the *netB*-positive strains that cause necrotic enteritis (NE) in poultry (7).

Bacterial strain typing is essential for outbreak investigations, epidemiological surveillance, and evaluation of control measures (8). Multilocus sequence typing (MLST) has been widely used for microbial genotyping and provides portable data easy for comparison among different laboratories (9). Owing to the significantly reduced costs of DNA sequencing over time, MLST has been extended to involve many hundreds of genes with the so-called core genome MLST (cgMLST) and whole-genome MLST (wgMLST) providing high resolution for optimal pathogen typing (10).

In C. perfringens, a cgMLST system was first utilized to describe the clonal relationship between *netF*-positive strains from enteritis cases in foals and dogs (11). A total of 1,349 genes were used to type 47 C. perfringens strains (11). The results showed that 32 *netF*-positive strains were classified into two clusters, including 26 and 6 strains, respectively (11). The scheme used in this previous study is not available on public databases for application. This underscores the need to standardize an accessible, genome-wide typing approach for a uniform and replicable characterization of C. perfringens. The aim of the current study was therefore to establish a publicly available cgMLST scheme for C. perfringens.

## RESULTS

### Definition and evaluation of C. perfringens cgMLST.

**FIG 1:**
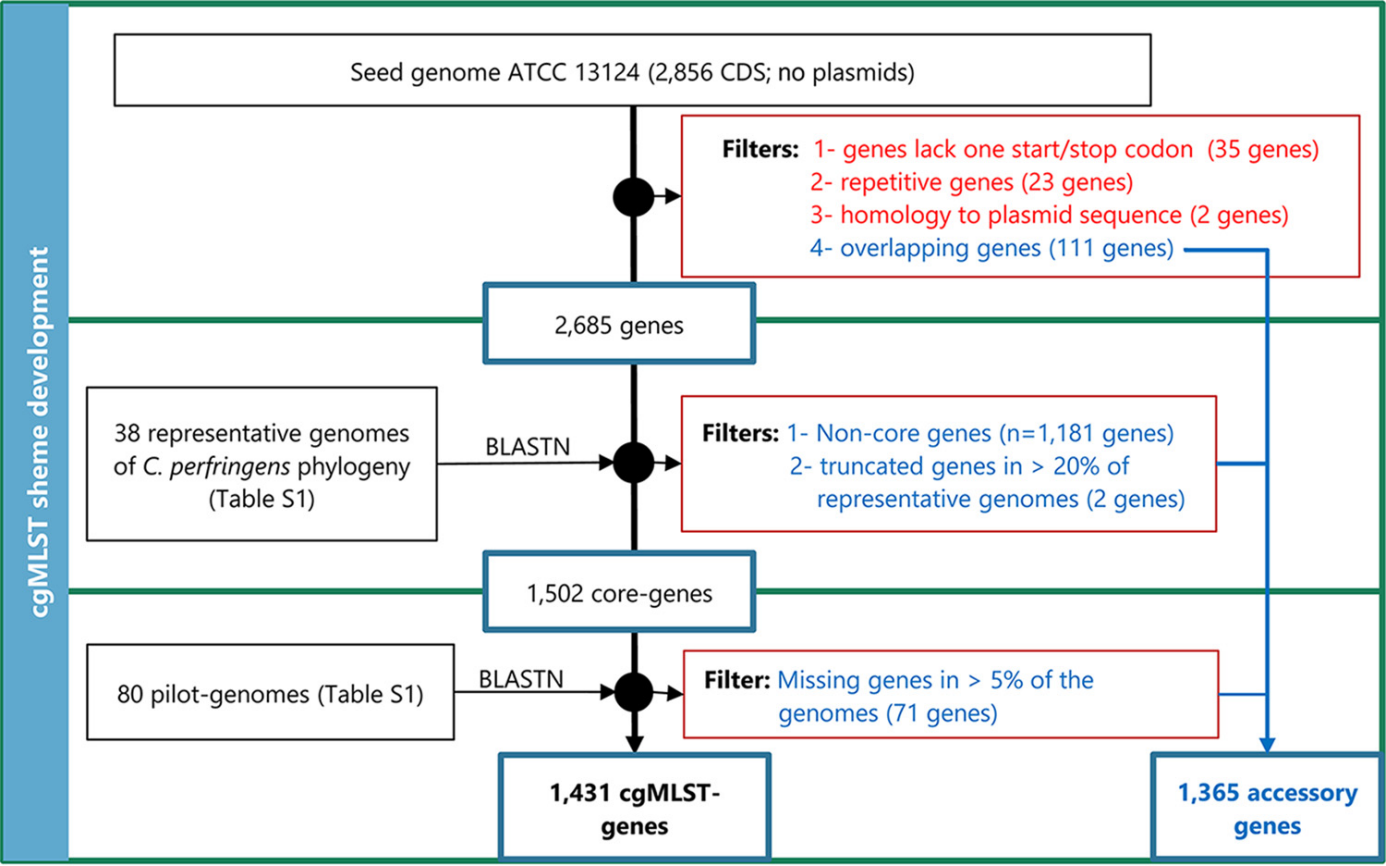
Schematic of the workflow to define the Clostridium perfringens core genome MLST targets.

As depicted in Fig. 1, the developed cgMLST scheme for C. perfringens included 1,431 target genes, corresponding to 50% of the genes with coding DNA sequences (CDS) in the reference genome ATCC 13124 (Table S1b in the supplemental material; Fig. S3A). The core target genes cover 41.2% (1.34 megabases) of the full genome size and were distributed unevenly across the genome, with a higher representation toward one chromosomal replichore (Fig. S3A). This distribution is consistent with previous studies that reported skewed presence of the species core genome (12, 13), possibly reflecting the uneven spread of mobile elements, particularly in C. perfringens genomes with chromosomal *cpe* (14). The length of the 1,431 target genes averaged 938.2 bp (standard deviation [SD], 578.1 bp; range, 84 to 4,350 bp) (Fig. S3D), with a mean ± SD GC content of 29.6 ± 3.1% (Fig. S3B).

In order to evaluate the novel scheme, an independent set of 282 C. perfringens genomes was used (Table S1a; Fig. S1). The cgMLST typing results showed that at least 95% of the cgMLST target genes were present in 273 of the 282 (96%) genomes examined (Table S1a; Fig. S4), with a mean ± SD of 99.08 ± 1.3% of the 1,431 target genes detected per genome for all genomes (Table S1a). The nontypeable target genes averaged 13 ± 20 genes (median, 7; range, 0 to 134) (Table S1a). This includes (i) genes that were completely absent from the genome, i.e., with BLAST hits below the defined thresholds (median, 2; mean, 8 ± 19 genes per genome), and (ii) genes without allele assignment due to frameshifts, internal stop codons, or absence of a single start/stop codon (median, 4; mean, 6 ± 6 genes per genome) (Table S1b).

The average number of alleles reported for each cgMLST target gene was 67 ± 28 alleles (range, 1 to 145) (Fig. S3C; Table S1b). The number of distinct cgMLST allelic profiles was 259 for the 282 genomes (missing data ignored in pairwise comparisons). The Simpson's index of the cgMLST was 0.999 (95% confidence interval, 0.999 to 1.0) (Table 1). Of interest, homologous recombination affected 930 of the 1,431 cgMLST genes (65%) as estimated by pairwise homoplasy index (PHI) statistic test (*P* < 0.05) (Table S1b).

**TABLE 1:** Discriminatory power and typing concordance of C. perfringens cgMLST and core genome SNPs

Typing method	No. of genotypes (out of 282 genomes)	Simpson's diversity index (95% confidence interval)	Adjusted Wallace index of concordance (95% confidence interval) of:		
			Core genome MLST	Core genome SNPs	Recombination-free core genome SNPs
Core genome MLST	259	0.999 (0.999–1.000)		0.758 (0.654–0.863)	1.000 (1.000–1.000)
Core genome SNPs	261	0.999 (0.999–1.000)	0.846 (0.718–0.974)		1.000 (1.000–1.000)
Recombination-free core genome SNPs	244	0.998 (0.997–0.999)	0.402 (0.254–0.551)	0.361 (0.261–0.461)	

### Comparison between cgMLST cluster analysis and SNP-based phylogeny.

To compare the typing results of cgMLST and sequence-based methods, we extracted the nucleotide sequences of the 1,431 cgMLST target genes from each genome in the evaluation set (282 genomes). A total of 151,338 variable sites (11.3%) were identified in an alignment concatemer of the 1,431 genes (1.34 Mb). Of these, 60,587 polymorphic sites were predicted with Gubbins to be outside recombinant regions, including 42,344 sites (3.1%) that were parsimony informative. The number of distinct genotypes defined with recombination-unfiltered single nucleotide polymorphisms (SNPs) was 261 (Simpson's index, 0.999) and with recombination-free SNPs was 244 (Simpson's index, 0.998), nearly equivalent to the distinct profiles identified with cgMLST (*n* = 259), indicating that cgMLST and core genome SNP provided comparable resolution for the 282 genomes (Table 1).

**FIG 2:**
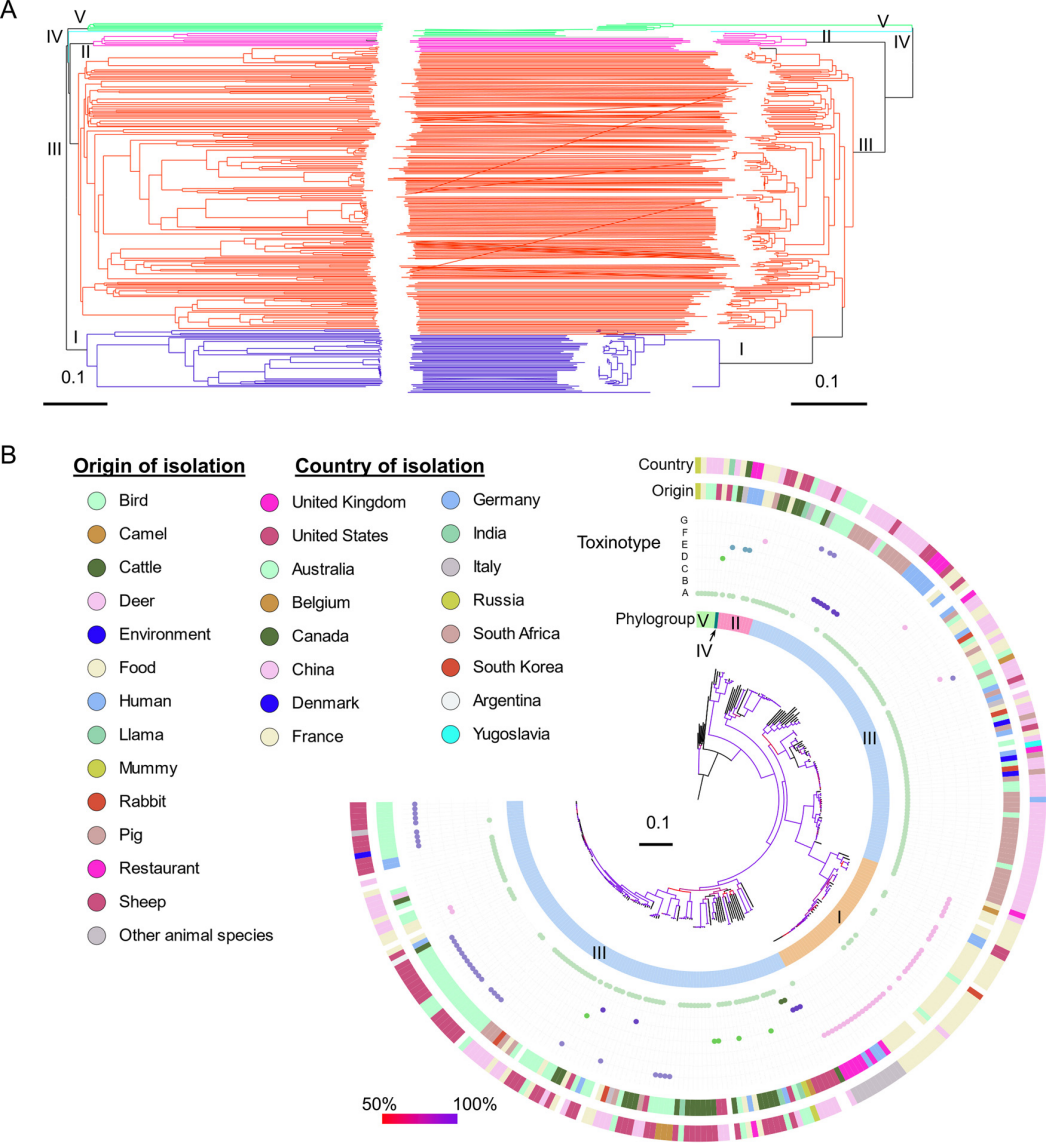
Comparison between typing results of allele- and nucleotide-based analysis of core genome MLST target genes. (A) Topological correspondence between the cgMLST neighbor-joining tree (left) and the SNP-based maximum-likelihood tree (right) is shown as a tanglegram. The branches of the tree were color-coded based on the species phylogroups indicated on the branches. The tanglegram was generated using Dendroscope v3.2.1027. (B) Phylogenetic maximum-likelihood tree based on nonrecombinant SNPs identified in the 282 Clostridium perfringens genomes. It shows the population structure of the species with the five phylogroups highlighted next to the ML tree, followed by the predicted toxin types of the strains and the origin and country of isolation as in the legend. The phylogenetic tree was visualized using iTOL v5 (49).

Sequence-based phylogenetic analysis of core polymorphic sites was also performed and compared with the clustering based on cgMLST allelic profiles (Fig. 2A). The resulting phylogenies were characterized by a high degree of topological congruence, with differences observed only in the deep branching structure of the trees (Fig. 2A). The five major phylogroups of C. perfringens (14, 15) were also well recognized by both methods. Clustering of the 282 genomes revealed that 77% of the genomes (*n* = 219) belonged to phylogroup III, followed by phylogroups I (*n* = 45), II (*n* = 11), IV (*n* = 1), and V (*n* = 6) (Fig. 2). As described (14), phylogroup I was mainly associated with humans, food, and food-related environments. Phylogroups II to V had a broad ecological distribution (Fig. 2B).

Furthermore, calculation of the number of differing alleles and nucleotides (SNPs) for each pair of genomes showed direct correlation (Fig. S5), except for large genetic differences between strains, where cgMLST distances became saturated and less informative (Fig. S5).

### Comparison of cgMLST and classical MLSTs.

Application of the three classical MLST schemes to 277 (of the 282) genomes required minor modifications; the *sigK* gene of the Deguchi scheme and the *recA* gene of the Jost and Hibberd schemes were included with reduced lengths (see Materials and Methods). In total, the Hibberd scheme had 11 loci of size 5,420 bp, the Deguchi scheme had 8 loci of size 5,090 bp, and the Jost scheme had 8 loci of size 2,833 bp. The sequence types (ST) defined with the modified schemes included 166 STs (Simpson's index, 0.989), 180 STs (Simpson's index, 0.993), and 191 STs (Simpson's index, 0.972) for Jost’s, Hibberd’s, and Deguchi’s schemes, respectively (Table 2). As expected, the MLST STs of the classical schemes could be further divided into different cgMLST profiles, indicating higher discrimination of cgMLST than the classical methods (Table 2). The tree topologies of the neighbor-joining (NJ) trees from the classical MLSTs were also less concordant with the cgMLST-based NJ tree (Fig. S6). The adjusted Wallace coefficient showed the highest agreement between classical and core genome MLST only when the cgMLST was used as the primary method for typing (Table 2).

**TABLE 2:** Discriminatory power and typing concordance of C. perfringens cgMLST and classical MLST schemes

Typing method	No. of genotypes (out of 277 genomes)	Simpson's diversity index (95% confidence interval)	Adjusted Wallace index of concordance (95% confidence interval) of:			
			Core genome MLST	Jost scheme	Hibberd scheme	Deguchi scheme
Core genome MLST	254	0.999 (0.999–1.000)		1.000 (1.000–1.000)	1.000 (1.000–1.000)	1.000 (1.000–1.000)
Jost scheme	166	0.989 (0.985–0.993)	0.070 (0.028–0.112)		0.650 (0.597–0.704)	0.558 (0.436–0.680)
Hibberd scheme	180	0.993 (0.990–0.995)	0.107 (0.047–0.167)	1.000 (1.000–1.000)		0.704 (0.604–0.805)
Deguchi scheme	191	0.993 (0.991–0.996)	0.115 (0.054–0.176)	0.919 (0.894–0.945)	0.755 (0.698–0.813)	

### Application of cgMLST in outbreak settings.

We applied the scheme to published outbreaks strains described by Carey and colleagues (16) and Kiu and colleagues (17). The first study (16) examined 92 strains from 76 patient and food samples by SNP calling and phylogenetic analysis. SNP calling was done by mapping sequencing reads to reference genomes using GalaxyTrakr's pipeline of the Center for Food Safety and Applied Nutrition (CFSAN) (18) and with NCBI Pathogen Detection, which uses genome assemblies and performs kmer-based clustering followed by 50-SNP single linkage clustering. Of the examined strains, 24 were from sporadic samples, and 16 were cultured from the same samples; hence, they were excluded from our reanalysis. The remaining 52 strains were from 13 retrospective foodborne outbreaks in New York, United States. The raw sequencing reads were recovered and *de novo* processed, revealing genomes with average size of 3.08 ± 0.21 Mb and average sequencing depth of 85.7 ± 18.6-fold (Table S1a).

**FIG 3:**
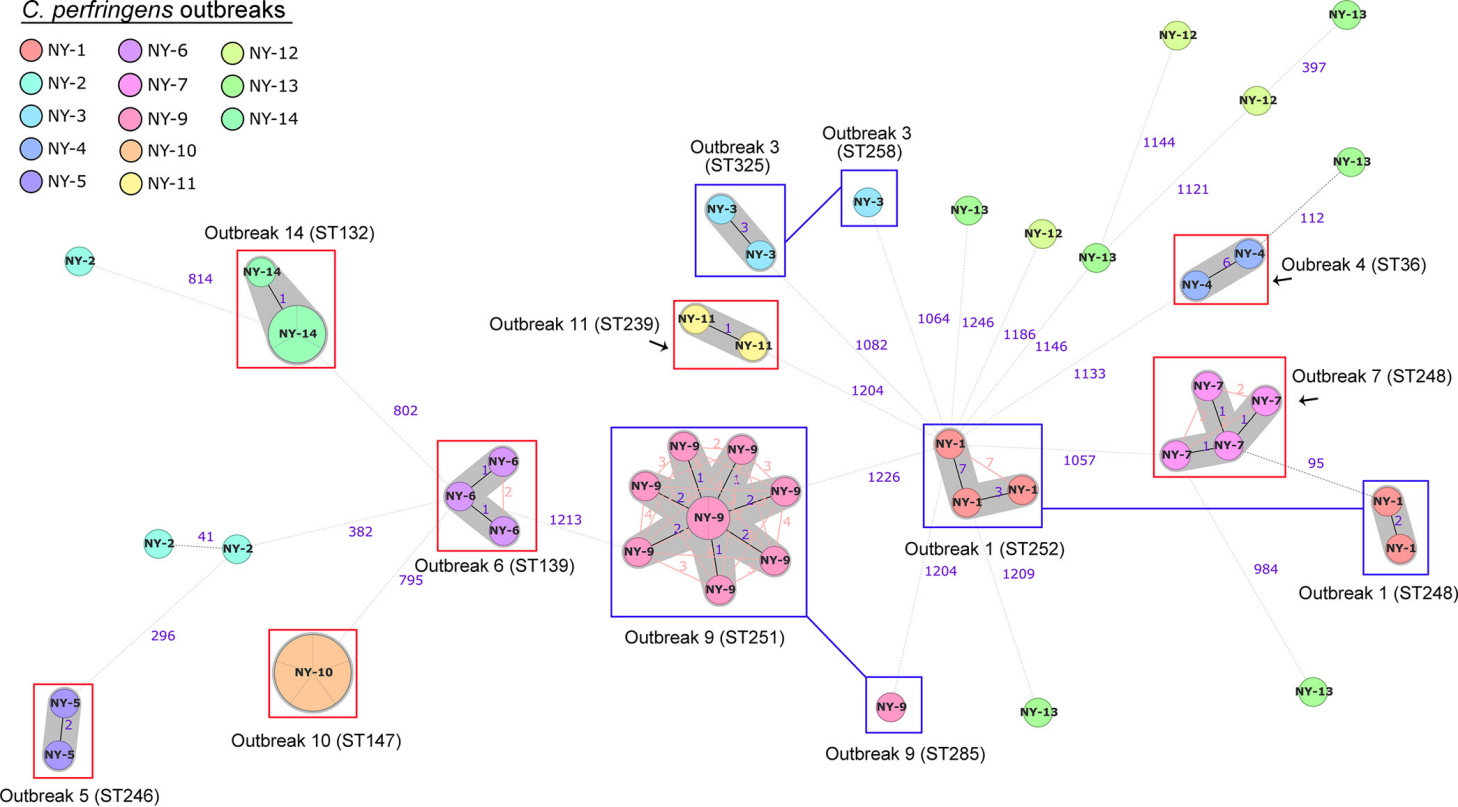
Minimum spanning tree based on the core genome MLST genes of 52 Clostridium perfringens strains from foodborne outbreaks in the United States. Nodes represent core genome MLST sequence types and were labeled according to the outbreak. Classical MLST types of the outbreak strains are also indicated where applicable. Red boxes mark monoclonal clusters detected in the outbreak. Connected blue boxes indicate more than one cluster identified in the outbreak. Clusters were identified based on fewer than seven allelic mismatches (gray shading). Numbers on the connecting lines illustrate the number of target genes with different alleles, represented by solid lines for allele differences below 40 and dotted lines for allele differences above 40. The minimum spanning trees was generated using Ridom SeqSphere v07 (https://www.ridom.de/).

The typeability percentage of the 52 genomes with cgMLST was 99.1% ± 0.4% on average (range, 97 to 99.8%; Table S1a), except one genome with low assembly quality having 91.3% of cgMLST targets. The 52 genomes were indexed into 45 distinct cgMLST allele profiles, with missing data ignored in pairwise comparisons (Fig. 3; Table S1d).

As shown in Fig. 3, strains from seven outbreaks (outbreaks 4, 5, 6, 7, 10, 11, and 14) were related by less than seven different alleles using cgMLST, possibly indicating a single strain cluster involved in each outbreak (Table S1d). Three other outbreaks (outbreaks 1, 3, and 9) included strains that were distributed in two clusters, with strains of each cluster having less than seven alleles different. The strain distribution in the two clusters indicated a possible dominance of one cluster in these outbreaks. One more outbreak (outbreak 2) involved 3 strains; 2 were related by 41 differing alleles and diverged from a third strain by 856 alleles (Table S1d). The remaining two outbreaks (outbreaks 12 and 13) involved distantly related strains; outbreak 12 had a mean of 1,269 ± 60 allele mismatches, whereas outbreak 13 had a mean of 1,286 ± 70 allele mismatches (Fig. 3; Table S1d). These results of the cgMLST were concordant with the published SNP analysis. In addition, classical *in silico* MLST typing with the Deguchi scheme (Fig. 3; Table S1d) showed that four strains in outbreak 7 and two strains in outbreak 1 were classified to the same ST 248, and two strains in outbreak 4 and one strain in outbreak 13 were also classified to ST 36 (Fig. 3; Table S1d). However, these outbreaks could be well distinguished into different genotypes with cgMLST, indicating better resolution of cgMLST to discriminate epidemiologically unrelated strains.

Kiu and colleagues (17) studied 109 C. perfringens strains from 14 foodborne outbreaks, 11 care homes-associated outbreaks, and 3 sporadic diarrheas in humans in England and Wales between 2011 and 2017. The phylogenetic analysis in that study was based on a maximum-likelihood (ML) tree of the Roary-calculated core gene alignment (19). We recovered the raw sequencing reads of 104 strains, representing outbreaks for which at least 2 strains were available per outbreak. Based on our assembly results, we further excluded one genome (GenBank accession no. ERR3377387) because of poor assembly metrics (only half of the genome was recovered). The other 103 genomes comprise 71 from 13 foodborne outbreaks and 32 from 10 care homes outbreaks. The genomes had a mean genome size of 3.31 ± 0.18 Mb with a mean sequencing depth of 86.03 ± 15.9-fold. On average, 99.1% (SD, 0.8%) of cgMLST targets were present in the genomes (range, 95.3 to 99.9%), with all genomes represented by 85 distinct cgMLST allele profiles.

**FIG 4:**
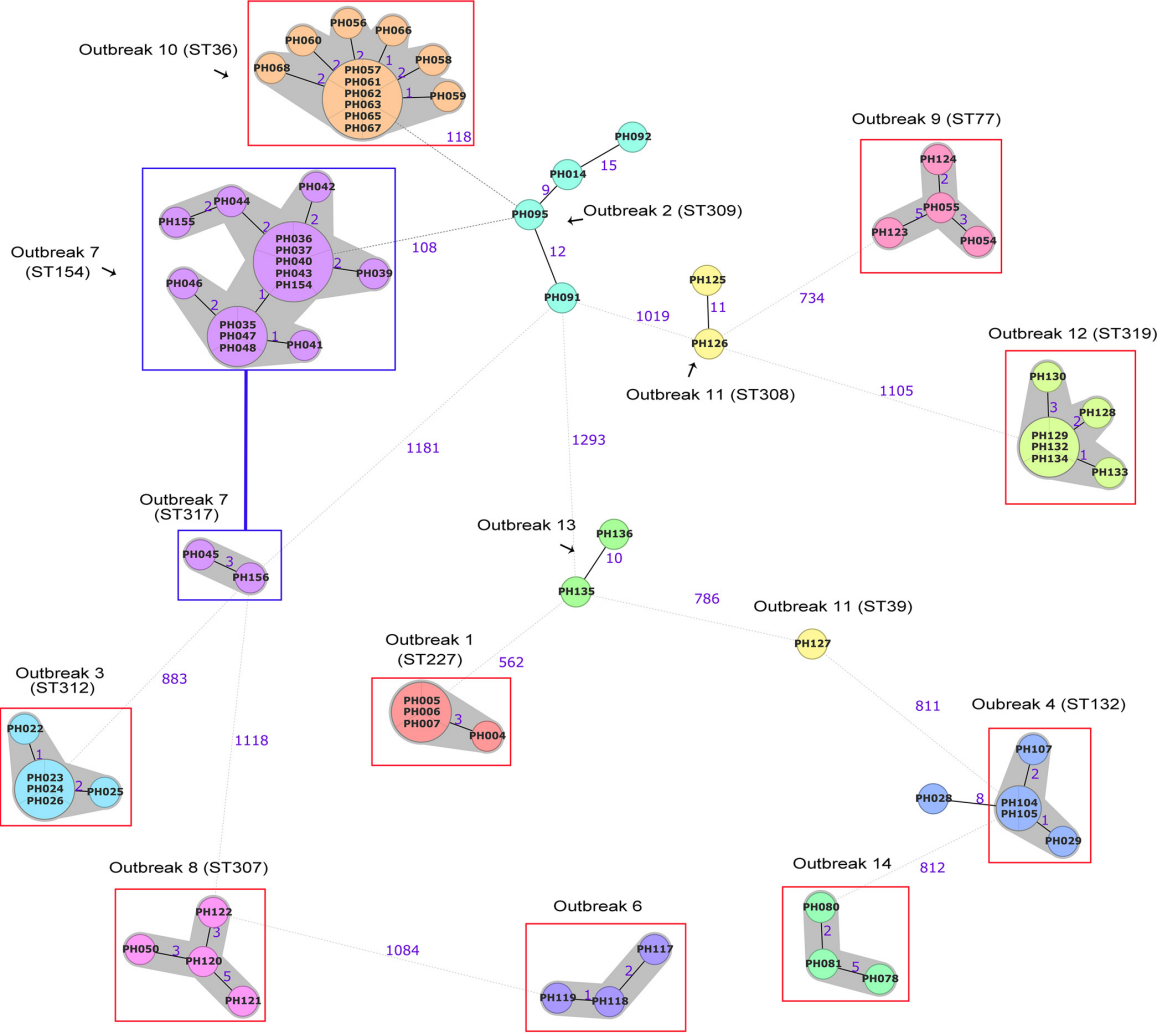
Minimum spanning tree based on the core genome MLST genes of 71 Clostridium perfringens strains from foodborne outbreaks in the United Kingdom. Classical MLST types of the outbreak strains are indicated where applicable. Nodes represent core genome MLST sequence types. Red boxes mark monoclonal clusters detected in the outbreak. Connected blue boxes indicate more than one cluster identified in the outbreak. Clusters were identified based on fewer than seven allelic mismatches (gray shading). Numbers on the connecting lines illustrate the number of target genes with different alleles, represented by solid lines for allele differences below 40 and dotted lines for allele differences above 40. The minimum spanning tree was generated using Ridom SeqSphere v07 (https://www.ridom.de/).

We first used cgMLST to reexamine the foodborne outbreaks (Fig. 4; Table S1e). Similar to the study of Carey and colleagues, we defined cgMLST clusters for strains that vary by at most seven alleles. While the 7-allele rule also worked well for these outbreaks and resulted in 8 (of the 13) outbreaks, each being represented by a single cgMLST cluster, there were some exceptions (Fig. 4; Table S1e). For example, outbreaks 4 and 13 contained strains that were up to 10 alleles apart; outbreak 11 had 3 strains, 2 of which were 11 alleles apart; and finally, outbreak 2 had strains that were 9 to 21 alleles apart (Table S1e). Another notable observation was outbreak 7, where two clusters of strains were observed, with a dominance of 1 cluster with 14 strains in one cluster versus 2 strains in the other small cluster. These results are consistent with the published SNP analysis in the original study, confirming that both methods can be robustly used to estimate the genetic distances at high resolution that is important for outbreak investigation.

**FIG 5:**
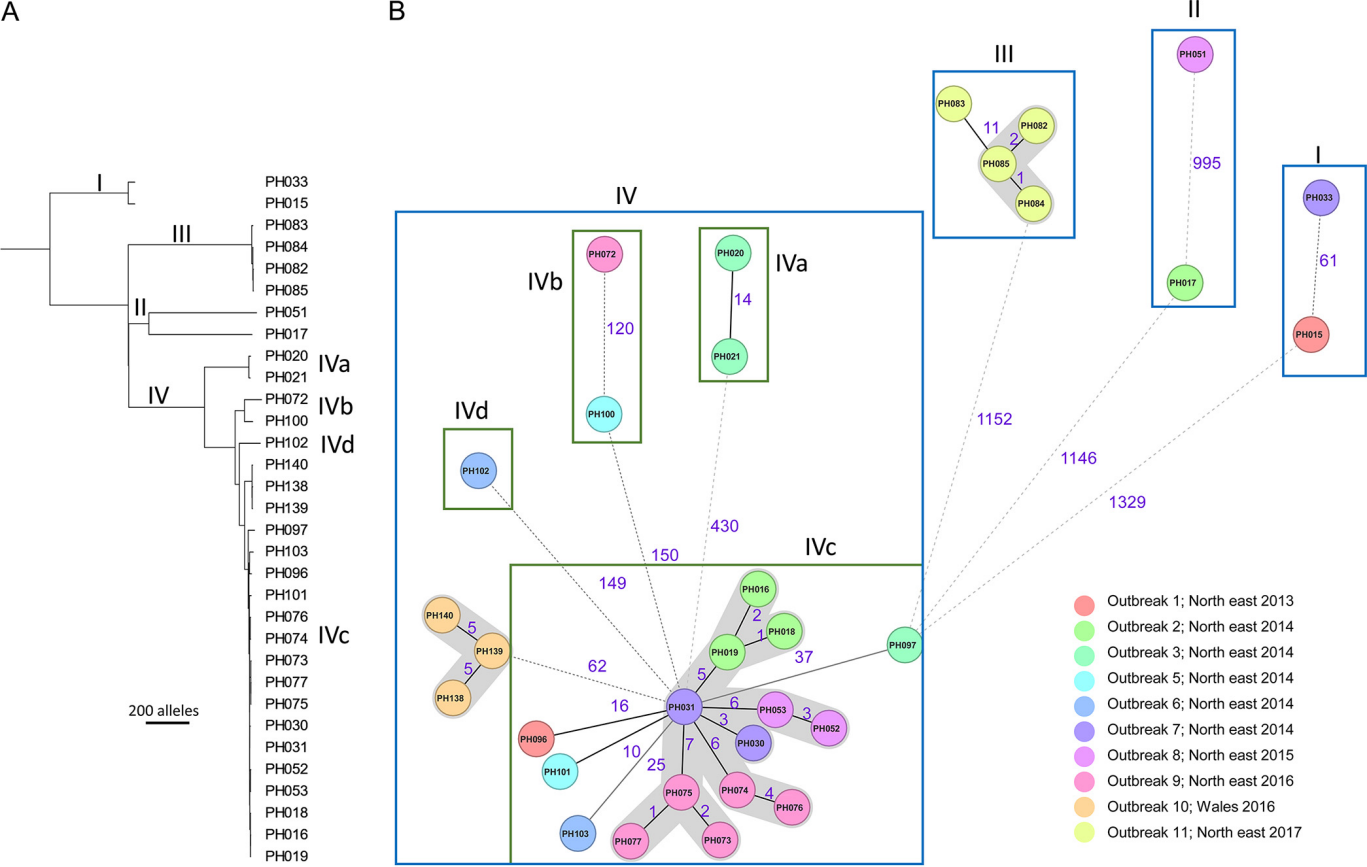
Investigation of 32 Clostridium perfringens strains from outbreaks in care homes in the United Kingdom. (A) Neighbor-joining tree of the 32 C. perfringens strains using the cgMLST allele typing system, with lineages identified based on SNP analysis highlighted as in the original study (17). (B) Minimum spanning tree using cgMLST of the same genomes. Lineages are indicated with boxes, and outbreaks are color-coded as in the legend. Clusters were identified based on fewer than seven allelic mismatches (gray shading). Numbers on the connecting lines illustrate the number of target genes with different alleles, represented by solid lines for allele differences below 40 and dotted lines for allele differences above 40. The minimum spanning tree was generated using Ridom SeqSphere v07 (https://www.ridom.de/).

The application of cgMLST to the 10 care homes-associated outbreaks was also consistent with the original study, showing that a clonal group of strains being spread in multiple outbreaks (named lineage IVc in the original study) (Fig. 5A; Table S1f). Using the cgMLST, the mean pairwise allelic diversity was 17 ± 12 alleles (range, 1 to 56 differing alleles) for this lineage, corresponding to a mean of 27 ± 15 SNPs (range, 2 to 68 SNPs). The results of cgMLST also reflect the published SNP analysis with respect to the observed high genetic diversity of strains involved in each of the care homes-associated outbreaks, with only few outbreaks involving a single strain cluster, such as outbreaks 10 (pairwise diversity 5.6 ± 0.6 alleles) and 11 (pairwise diversity 7 ± 5.5 alleles) (Fig. 5B; Table S1f).

## DISCUSSION

Whole-genome sequencing represents a powerful molecular epidemiological tool for pathogen subtyping and outbreak investigations. In this study, we describe a cgMLST scheme for C. perfringens based on 1,431 highly conserved core genes. This novel scheme has several advantages over the previous cgMLST of Gohari et al. (11). The setup of our scheme included a core genome defined by representatives of all phylogenetic groups of the species (Fig. S2), as detailed very recently (14, 15). In addition, we implemented a further refinement step of the scheme with 80 pilot genomes to improve the typing output results (Fig. 1). The developed scheme also included more genes than the previous scheme (11) and was evaluated on a larger number of genomes. This novel scheme was also made publicly available with the establishment of a nomenclature server on Ridom website (https://www.cgmlst.org/ncs) for use with SeqSphere (20) and PubMLST (https://pubmlst.org/) as an open-source platform that provides web-accessible analyses for comparative genomics (21, 22). The publicly accessible scheme and allele nomenclature server are expected to facilitate comparison of MLST typing results between laboratories and enable the establishment of consistent nomenclature, which is essential for global epidemiological surveillance and rapid communication of outbreak data (10). Furthermore, it is a valuable resource for small laboratories with limited computer resources and bioinformatics expertise.

The cgMLST system has the advantage that the resulting data are lightweight and easily portable, allowing sharing of typing results via centralized online databases. Decentralized, standalone workflows of cgMLST using allele hashing have also been described (23, 24), which are useful for studying sensitive outbreak data. In addition, the analytical workflow of cgMLST is less computationally intensive once *de novo* genome assembly is available, which is routinely performed in practice for sequenced bacterial genomes. The tools used for cgMLST typing are often user-friendly and do not require complex bioinformatics expertise, and the underlying methodology does not involve preselection of an appropriate (i.e., phylogenetically related) reference for the data set under study, which is important for reference-based SNP typing methods. The allele nomenclature databases of cgMLST are easily expandable with new typing data that enhance global epidemiologic surveillance. On the other hand, because cgMLST uses assembled genomes for typing, the results are likely to be affected by sequencing errors and assembly artifacts that are difficult to distinguish from true variants (25). The high sequencing depth may, however, help improve the accuracy and reproducibility of cgMLST calls. In addition, the quality of DNA and sequencing libraries, as well as the purity of cultured isolate, are also important for correct typing with cgMLST. The cgMLST system can, moreover, be used as an indicator for genome quality by determining the percentage of typeable targets.

Application of the cgMLST scheme to 282 C. perfringens genomes and comparison with core genome SNP typing revealed a high degree of congruence in typing results with comparable discriminatory power based on the Simpson’s index (Table 1). The cgMLST also proved useful for inferring the species population structure and defining the five major phylogenetic groups (Fig. 2). Nevertheless, it must be noted that the analysis of nucleotide sequences of core genes provided better resolution in the deep-branching structure of the inferred phylogeny. This can be explained by the fact that cgMLST distances were not directly correlated with SNP variations when genetic differences between the strains were large (Fig. S5), possibly because the cgMLST collapses multiple SNP variations of genes into numbered alleles and has an upper limit of allelic differences equal to the total number of genes in the scheme. In addition, the tree derived from the core genome SNPs, which was based on nucleotide alignments followed by a maximum-likelihood phylogenetic method, provides a more robust phylogenetic framework of isolate data than the distance-based neighbor-joining methods from the cgMLST allele data (26).

Studies have reported different cluster thresholds for cgMLST typing of different bacterial species. These are usually less than 10 alleles between epidemiologically linked strains, which may be caused by putative microevolutionary events (27). Here, we used a cutoff threshold of 7 allelic differences as a starting point for studying C. perfringens-associated outbreaks. With this threshold, the cgMLST cluster results were consistent with published SNP analyses, indicating the often-nonexclusive presence of a clonal group of C. perfringens strains in the outbreaks, where there is always the possibility of multiple clones or unrelated strains being detected in an outbreak. This represents either a commensal strain isolated by chance or the involvement of multiple clones of strain populations in these outbreaks. Although the cutoff a seven-alleles distance was consistent with the epidemiologic background information for the foodborne outbreaks in the United States, it did not fit all foodborne outbreaks from the United Kingdom, where the clonal group allelic distance of the outbreak strains varied by up to 21 alleles, e.g., in outbreak 2. The fact that C. perfringens has a highly variable genome, with ∼65% of cgMLST targets significantly affected by homologous recombination, may have complicated the determination of an appropriate cutoff for future elucidation of infection chains. Therefore, it is necessary that the cgMLST clusters are interpreted along with epidemiological data. Taken together, the cgMLST clustering results provided a comparable resolution to previously published SNP-based analysis and led to similar conclusions to those in the original studies.

In summary, this study describes the successful establishment of a cgMLST scheme for high-resolution molecular typing of C. perfringens. The scheme and an allele nomenclature database are publicly available for broad and standard epidemiological studies. We have shown that the typing results of cgMLST are highly comparable to core genome SNP typing and more discriminative than classical methods. Finally, we demonstrated the usefulness of the novel cgMLST scheme for outbreak investigations by reproducing the results of previous SNP analysis reports and reaching the same conclusions.

## MATERIALS AND METHODS

### C. perfringens cgMLST scheme definition.

Figure 1 visualizes the workflow used to define the cgMLST targets. For scheme development, we used 80 genomes (see Table S1a and Fig. S1 in the supplemental material). The genome of the type strain was used as a reference (ATCC 13124; GenBank accession no. NC_008261.1 as of 3 May 2020), and a further 38 genomes were selected as representative of the five major phylogroups of the species (14, 15), including 26 fully circularized and 12 draft genomes (see Table S1a; Fig. S1 and S2). We implemented a three-step procedure to develop the scheme (Fig. 1). First, the cgMLST target definer v1.5 function of SeqSphere+ v7.1.0 (20) was used in standard mode to remove 171 genes from the reference genome based on repetition, overlapping, or truncation. In addition, two reference genes with at least 90% sequence identity to C. perfringens plasmid sequences were excluded. Second, BLAST v2.2.12 (28), as implemented in cgMLST target definer v1.5 (20), identified 1,181 noncore genes in the 38 representative genomes and the reference strain (>90% identity and 100% overlap), which were removed along with two genes that were incomplete in more than 20% of these genomes (Table S1a; Fig. 1; Fig. S2). Third, using the filtered 1,502 genes, we performed allele typing to the 80 C. perfringens genomes with SeqSphere+ v7.1.0 with at least 90% sequence identity and 99% overlap to the reference genes. Only complete genes were assigned to allele numbers, i.e., alleles were not assigned to genes with frameshifts or in-frame stop codons or carry non-GATC characters. In-frame multiple insertions or deletions (indels) were allowed up to three codons per gene relative to the reference genes. The typeability percentage was estimated in this pilot set of genomes (Table S1a), and we excluded 71 genes that were missing or have not been typed in more than 5% of these genomes. The final set of core genes served as targets for cgMLST (Table S1b). In addition, 1,365 genes were compiled from the filtered genes as accessory targets (Table S1c; Fig. 1). The pairwise homoplasy index (PHI) was calculated using PHIPack as a statistical method for detecting recombination in cgMLST targets (Table S1b).

### Evaluation of C. perfringens cgMLST targets.

For scheme evaluation, an independent set of 282 C. perfringens genomes was compiled from the National Center for Biotechnology Information Reference Sequences (RefSeq) database and recent studies (14, 15, 29) excluding duplicates, genomes with less than 95% average nucleotide identity (ANI) using the FastANI algorithm and genomes previously used in the scheme setup (Table S1a, Fig. S1). For the raw sequencing reads (Table S1a), the sequence quality was checked with FastQC (30) followed by genome assembly using shovill v1.0.9 (31) with activated flags for read trimming with Trimmomatic (32) and filtering contigs based on a kmer coverage threshold of 5-fold and a minimum contig length of 500 bp. For all genomes, FastANI v1.3 (33) calculated the ANI values compared to the reference genome (ATCC 13124), QUAST v4.3 (34) assessed the quality of genome assemblies, and CheckM v1.1.3 (35) estimated levels of genome completeness, contamination, and heterogeneity. The 282 genomes were then scanned for the presence of cgMLST genes with BLAST at nucleotide identity >90% and alignment coverage >99%. Allele calling was done with SeqSphere+ v7.1.0 (20), and the resultant profiles were clustered, with missing data being ignored in pairwise comparisons.

### Comparison between core genome MLST and SNP typing.

Phylogenetic analysis of core genome single nucleotide polymorphisms (SNPs) was performed using nucleotide sequences of all cgMLST genes from the evaluation set (282 genomes) (Table S1a; Fig. S1). For each gene, multiple sequence alignment was done with MAFFT v7.30 (36, 37). The resulting gene alignments were concatenated in a supermatrix core genome alignment. Next, we identified polymorphic sites in the cgMLST genes and used Gubbins v2.2.1 (38) in default mode to mask putative recombination regions. For Gubbins, a pseudo-whole-genome alignment was prepared as described (39) using the reference genome ATCC 13124 labeled with SNP sites of cgMLST genes for each sample. RAxML v8.2.10 was used to generate a maximum-likelihood (ML) phylogenetic tree using the general time-reversible (GTR)-gamma model and 100 bootstrap replicates (40). Topological concordance between cgMLST-based neighbor-joining (NJ) tree and SNP-based ML tree was visualized using the tanglegram algorithm (41) in Dendroscope v3.2.1027 (42). Furthermore, the Simpson's diversity index (43) and the adjusted Wallace index of concordance (44) were calculated using the comparing partitions tool (45) to compare the discrimination levels and assess the congruence of the typing results, respectively.

### Comparison between cgMLST and classical MLST.

Three classical MLST schemes previously described for C. perfringens were used to compare with cgMLST (46–48). The MLST genes were *in silico* extracted from the genomic data with BLASTN v2.9.0 (28), including *plc*, *groEL*, *gyrB*, *nadA*, *pgk*, *sigK*, *sodF*, and *colA* described by Deguchi et al. 2009 (47) (later also referred to as Deguchi scheme); *plc*, *ddlA*, *dut*, *glpK*, *gmk*, *recA*, *sod*, and *tpiA* described by Jost et al. (46) (later also referred to as Jost scheme); and *plc*, *ddlA*, *dut*, *glpK*, *gmk*, *recA*, *sod*, *tpi*, *dnaK*, *gyrA*, and *groEL* described by Hibberd et al. (48) (later also referred to as Hibberd scheme). In the Deguchi scheme, 405 bp of *sigK* gene were included instead of 589 bp because of gene truncation in 17 (out of 282) genomes. For Hibberd’s and Jost’s schemes, we also included 208 bp of *recA*, as 16 genomes had this gene fragmented at contig breaks or had the gene split by an insertion of an ∼2,860-bp DNA region. In addition, 5 genomes were excluded because of missing MLST loci; thus, 277 genomes were left for comparison. Extracted MLST loci of each genome were aligned using MAFFT v7.30 (36, 37) and assigned allele numbers with SeqSphere+ v7.1.0. The topological congruence of NJ trees from cgMLST and classical MLSTs was visualized with the tanglegram algorithm (41). Simpson's diversity index (43) and adjusted Wallace coefficient (44) were calculated as mentioned earlier.

### Application of cgMLST in outbreak settings.

To evaluate the applicability of the C. perfringens cgMLST scheme for outbreak analysis, we reanalyzed published genomic data of C. perfringens strains from foodborne outbreaks in the United States and United Kingdom, as well as outbreaks in care homes in the United Kingdom (Table S1a). The genome sequences from these outbreaks were recently analyzed using SNP approaches (16, 17) and made publicly available along with the associated metainformation. In this study, the raw sequencing data were downloaded and *de novo* processed using FastQC (30), shovill (31), FastANI (33), Quast (34), and checkM (35) as mentioned above. The cgMLST scheme was applied, and minimum spanning trees (MST) based on cgMLST allelic profiles were produced using SeqSphere+ v7.1.0, ignoring missing loci in pairwise comparisons.
