# Supplementary material for: Establishment of a Publicly Available Core Genome Multilocus Sequence Typing Scheme for Clostridium perfringens
Source: Microbiol Spectr. 2021 Oct 27;9(2):e00533-21. doi: 10.1128/Spectrum.00533-21 (PMC8549748; doi:10.1128/Spectrum.00533-21)
Supplement: SUPPLEMENTAL FILE 2 — Supplemental material. Download SPECTRUM00533-21_Supp_2_seq12.pdf, PDF file, 1.2 MB [file spectrum00533-21_supp_2_seq12.pdf]

**Supplemental Material:** Supplemental Figures S1 – S6 and legends of Supplemental Table S1

# Establishment of a publicly available core genome multilocus sequence typing scheme for *Clostridium perfringens*

Mostafa Y. Abdel-Glil,[a,b#] Prasad Thomas,[a*] Jörg Linde,[a] Keith A. Jolley,[c] Dag Harmsen,[d] Lothar H. Wieler,[e] Heinrich Neubauer,[a] Christian Seyboldt[a]

[a]Institute of Bacterial Infections and Zoonoses, Friedrich-Loeffler-Institut, Naumburger Str. 96A, 07743 Jena, Germany.

[b]Department of Pathology, Faculty of Veterinary Medicine, Zagazig University, Sharkia province, Egypt.

[c]Department of Zoology, University of Oxford, Oxford, United Kingdom.

[d]Department of Periodontology and Operative Dentistry, University Hospital Muenster, Muenster, Germany

[e]Robert Koch-Institut, Nordufer 20, 13353, Berlin, Germany.

[*]Present address: Division of Bacteriology and Mycology, ICAR-Indian Veterinary Research Institute, Izatnagar, 243122, India

**Supplemental Figures S1 – S5**

---

Figure S1: Genome sets used for the development and evaluation of the cgMLST scheme.

Figure S2: Representativeness of the selected 38 genomes to *Clostridium perfringens* phylogroups.

Figure S3: Characteristics of the 1,431-core genome MLST genes.

Figure S4: Performance of the 1,431-core genome MLST scheme on the evaluation set of 282 genomes.

Figure S5: Correlation between the numbers of pairwise SNP distances to the pairwise cgMLST allelic differences.

Figure S6: Topological concordance between the neighbor-joining trees from cgMLST and classical MLST methods for *C. perfringens* is represented as tanglegram.

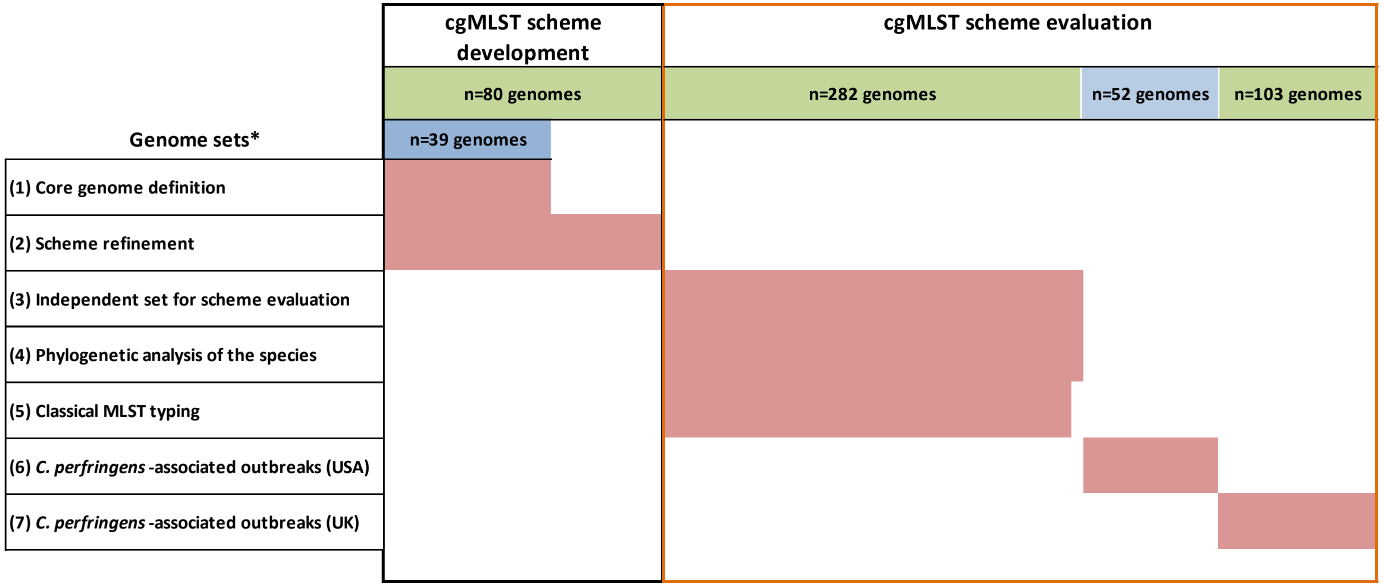


**Figure S1: Genome sets used for the development and evaluation of the cgMLST scheme.**
*the genome sets used as follow:
(1): 39 genomes were used for core genome definition, including the reference genome and 38 representative genomes of the five major phylogenetic groups of the species as depicted in Figure S2 and described in detail in previous studies by Abdel-Glil et al., 2021 and Feng et al., 2020.
(2): 80 genomes were used to initially evaluate the core genome targets and further exclusion of target genes that were missing or have not been typed in more than 5% of these genomes.
(3): 282 genomes collected from the NCBI Refseq database and independent studies were used for the evaluation of the developed scheme.
(4): the 282 genomes that were used for scheme evaluation were also used for SNP calling and phylogenetic analysis.
(5): classical typing with MLST was done for 277 out of the 282 genomes. The excluded five genomes had missing MLST loci.
(6) 52 *C. perfringens* genomes from foodborne outbreaks in the USA were processed with the cgMLST scheme.
(7) 103 *C. perfringens* genomes from foodborne outbreaks and outbreaks in care homes in England and Wales were processed with the cgMLST scheme.

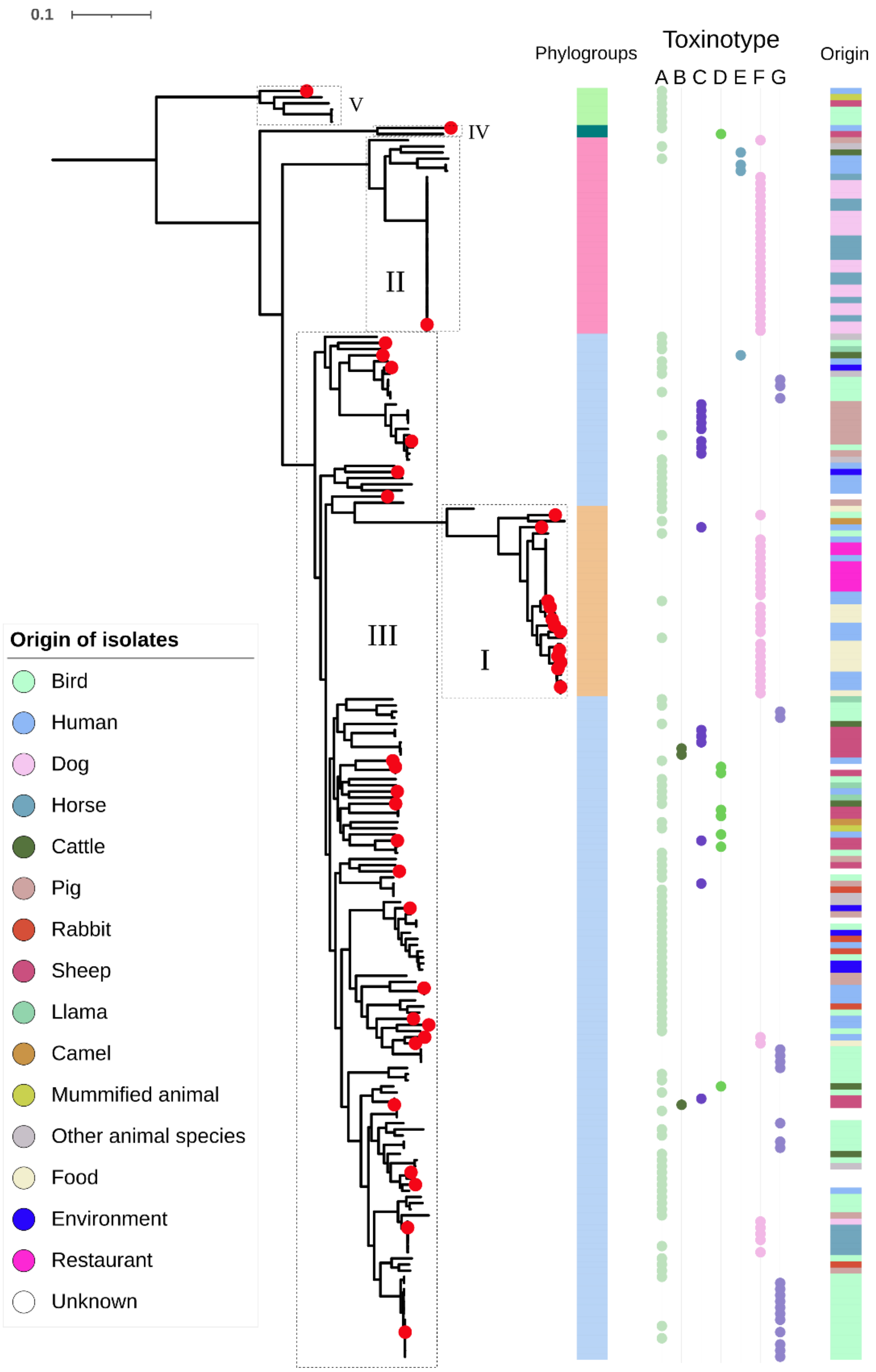


**Figure S2: Representativeness of the selected 38 genomes to *Clostridium perfringens* phylogroups.** Phylogenetic maximum likelihood tree based on 206 *C. perfringens* genomes as previously described (1). Selected genomes used to calculate the species core genome are indicated by red color-coding at the tips of tree branches. Dashed boxes numbered I to V, indicate the five phylogroups of *C. perfringens*, also highlighted next to the ML tree, followed by the predicted toxin types of the strains and the host origin of isolation as in the legend.

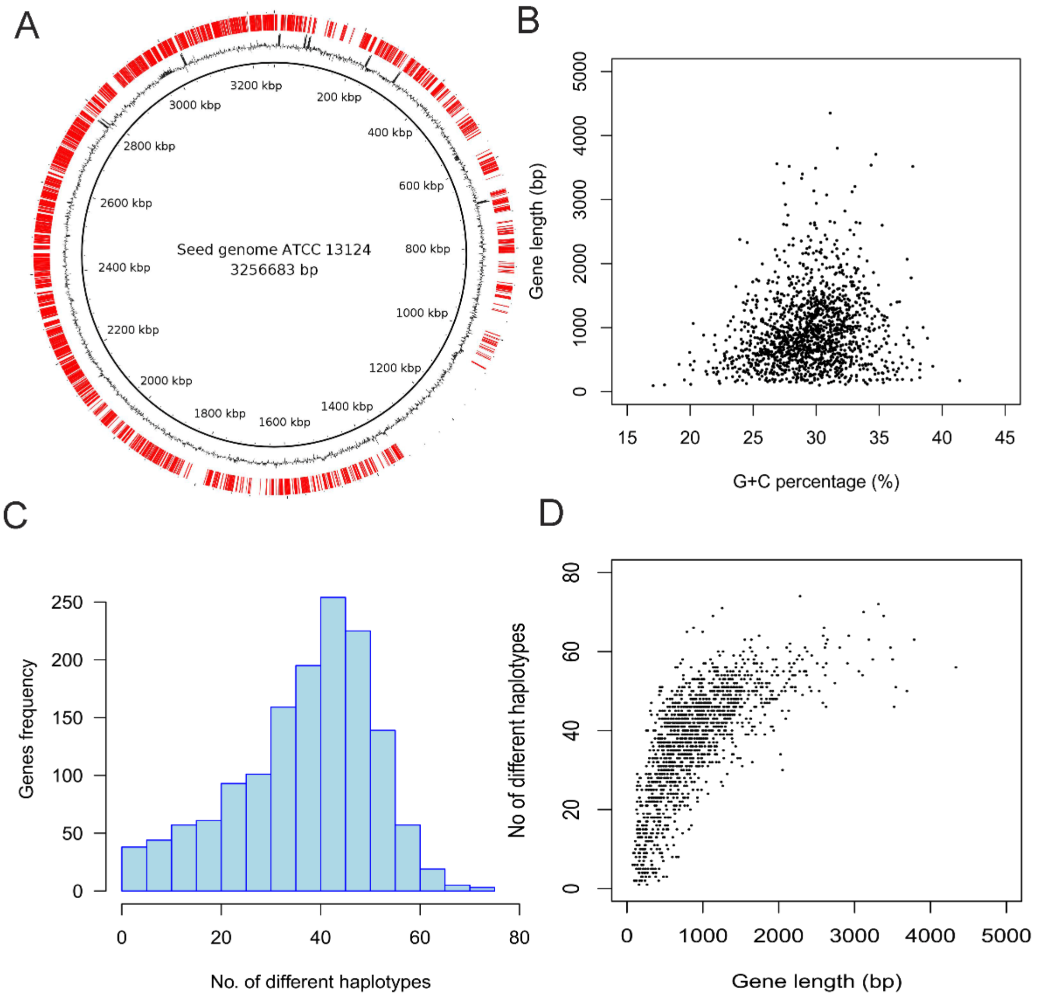


**Figure S3: Characteristics of the 1,431-core genome MLST genes.** (A) The distribution of the cgMLST targets (red, outer circle) across the *Clostridium perfringens* ATCC 13124 reference genome. Inner circles represent the G+C content and genomic positions, respectively. (B) Plot showing the lengths and G+C content of the cgMLST targets. (C) Plot showing the frequency of different allelic variants observed for each of the cgMLST targets. (D) Plot showing a direct association between the length of cgMLST target genes and the number of variants detected for each gene.

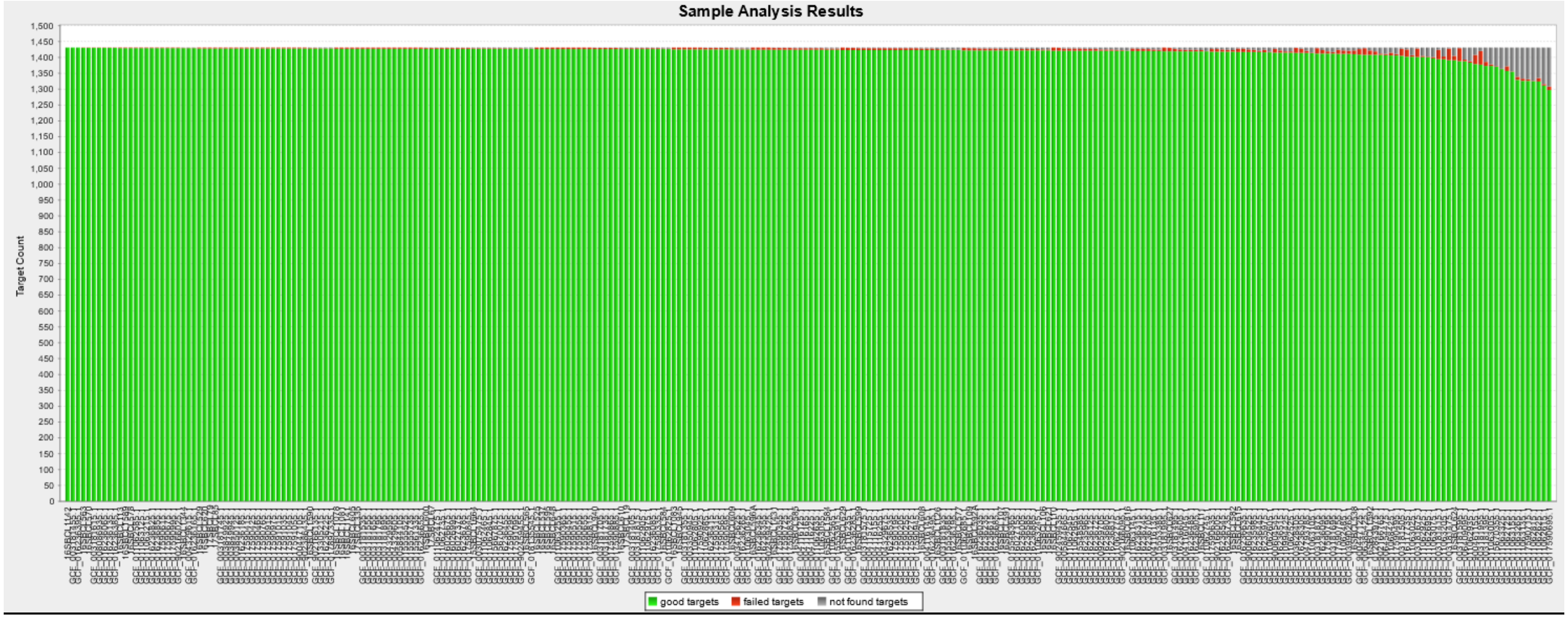


**Figure S4: Performance of the 1,431-core genome MLST scheme on the evaluation set of 282 genomes.** The evaluated genomes are represented by columns and the typing results of each cgMLST target gene are presented in the rows, with green, red, and grey coloration indicating genes typed and assigned allele number, genes not typed due to failed quality metrics (e.g. gene with frameshift), and genes not typed because insufficient BLAST hit, respectively.

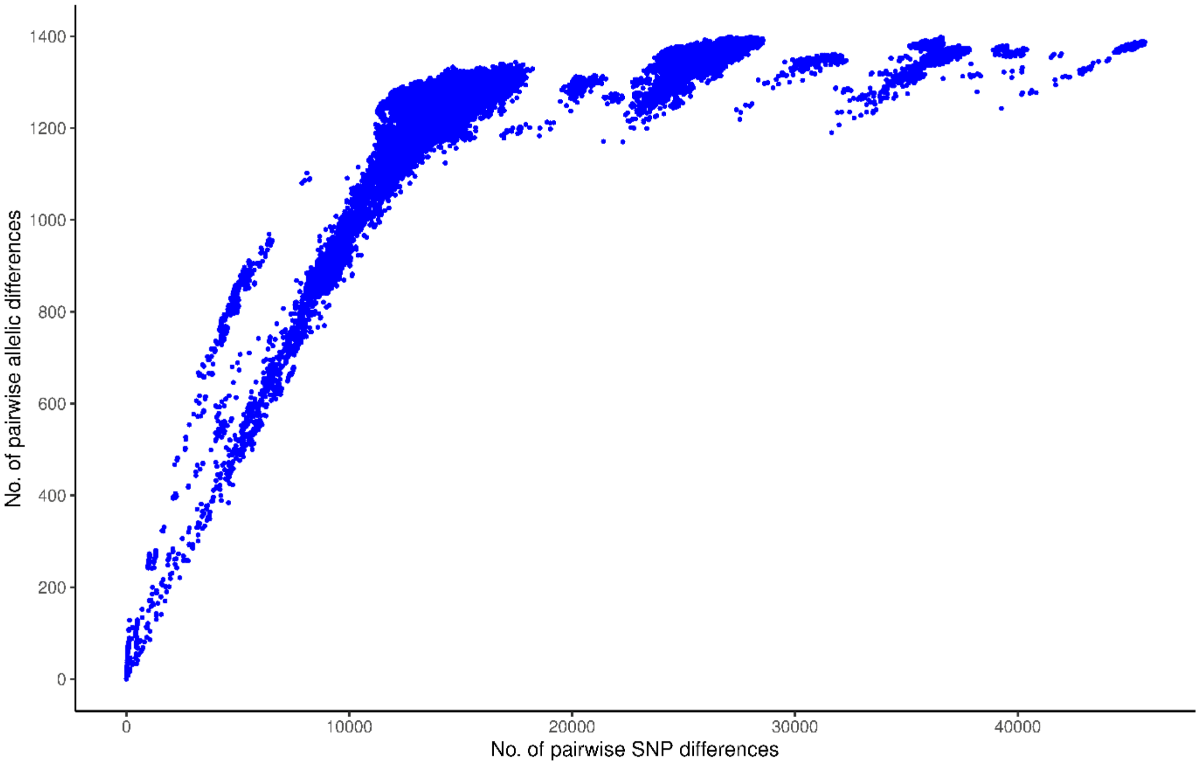


**Figure S5: Correlation between the numbers of pairwise SNP distances to the pairwise cgMLST allelic differences.**

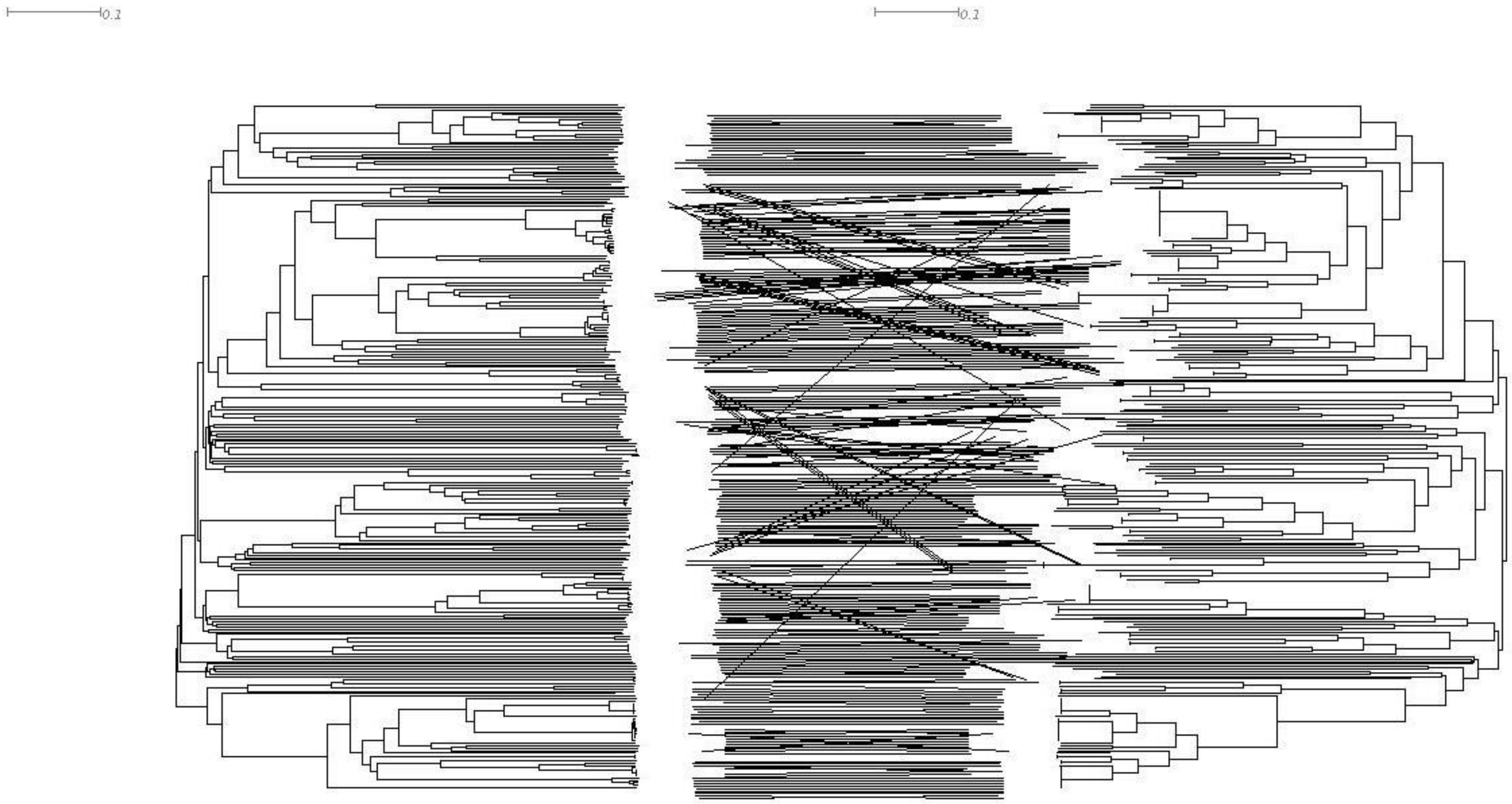


**Figure S6A: Topological concordance between the neighbor-joining (NJ) trees from cgMLST (left) and classical MLST methods for *C. perfringens* (right) is represented as tanglegram.** The NJ tree from the classical MLST scheme of Jost et al., (2006) (2) (Right) was compared to the NJ tree calculated from the cgMLST (left). The trees were mid-point rooted

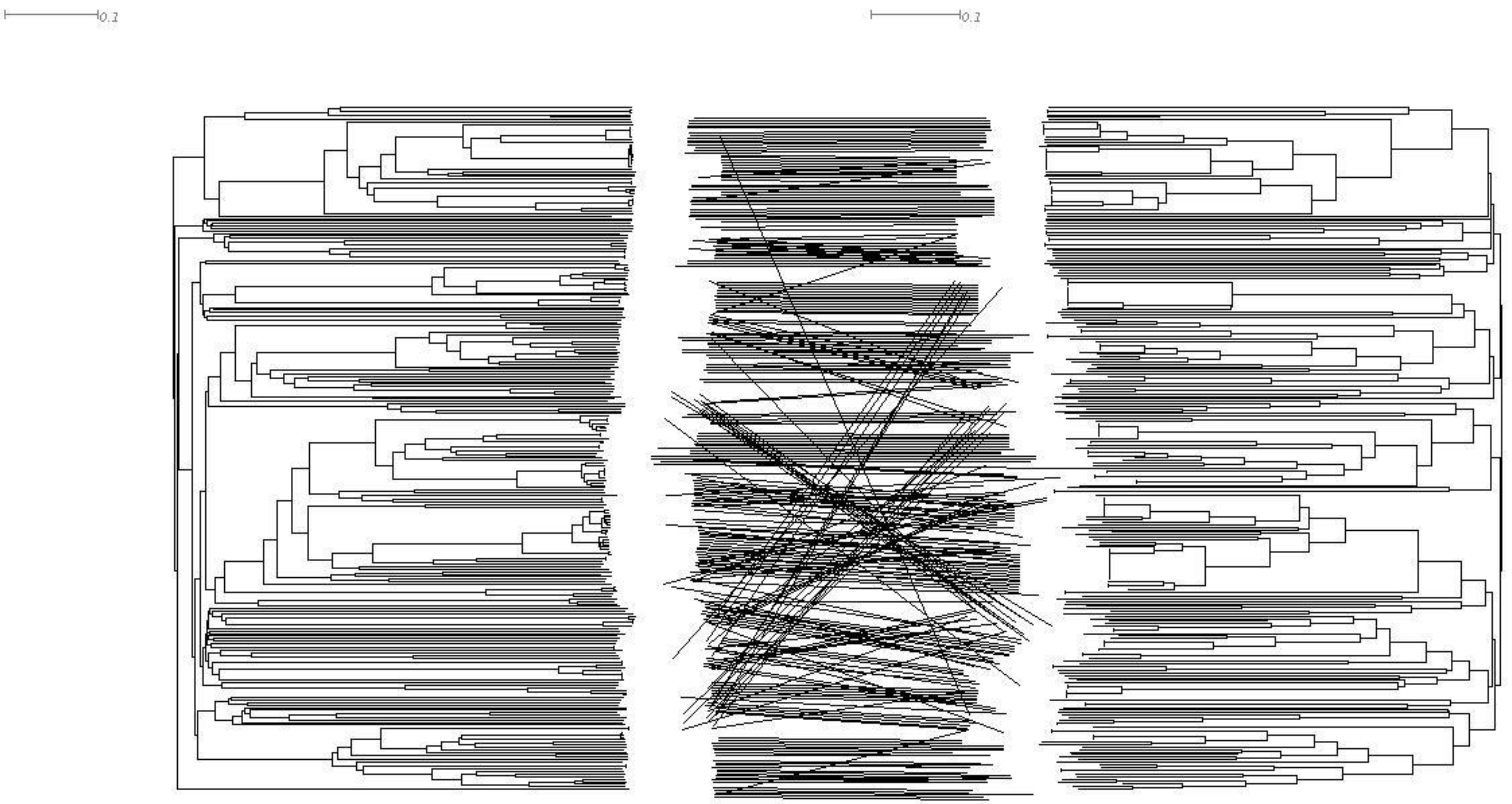


**Figure S6B: Topological concordance between the neighbor-joining (NJ) trees from cgMLST (left) and classical MLST methods for *C. perfringens* (right) represented as tanglegram.** The NJ tree from the classical MLST scheme of Deguchi et al., (2009) (3) (right) was compared to the NJ tree calculated from the cgMLST (left). The trees were mid-point rooted.

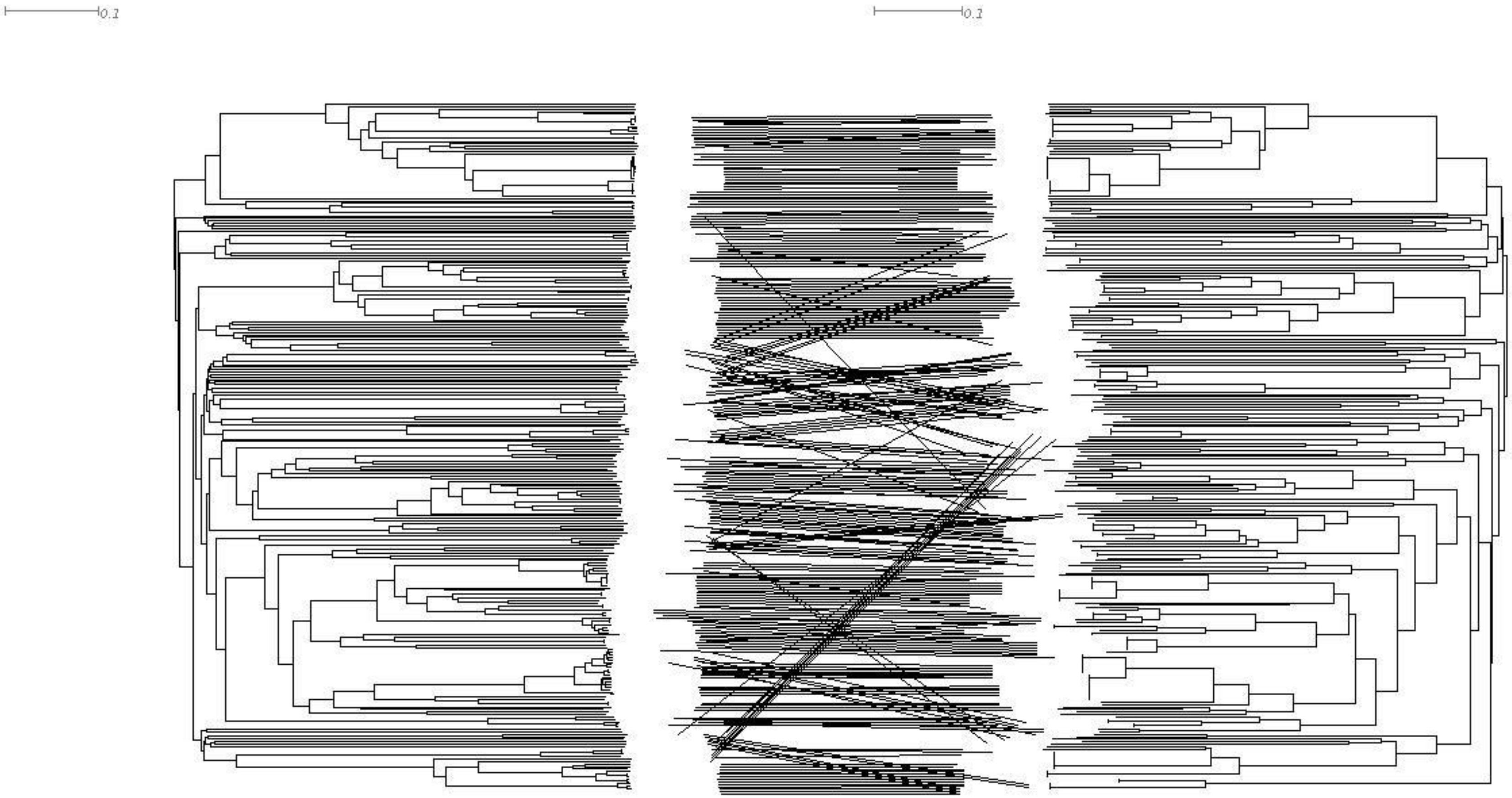


**Figure S6C: Topological concordance between the neighbor-joining (NJ) trees from cgMLST (left) and classical MLST methods (right) for *C. perfringens* represented as tanglegram.** The NJ tree from the classical MLST scheme of Hibberd et al., (2011) (4)(right) was compared to the NJ tree calculated from cgMLST (left). The trees were mid-point rooted

**Legends of Supplemental Tables S1a to S1f**

Table S1a: Details of *C. perfringens* genomes used for cgMLST scheme development, evaluation, and application.

Table S1b: Characteristics of the 1431 core genome multilocus sequence typing genes.

Table S1c: 1,365 Clostridium perfringens accessory target genes.

Table S1d: Pairwise cgMLST allelic differences between the genomes of 52 *C. perfringens* strains from 13 foodborne outbreaks in the USA.

Table S1e: Pairwise cgMLST allelic differences between the genomes of 71 *C. perfringens* strains from foodborne outbreaks in the UK.

Table S1f: Pairwise cgMLST allelic differences between the genomes of 32 *C. perfringens* strains from outbreaks in the care homes in the UK.

References


1. Abdel-Glil MY, Thomas P, Linde J, Busch A, Wieler LH, Neubauer H, Seyboldt C. 2021. Comparative in silico genome analysis of *Clostridium perfringens* unravels stable phylogroups with different genome characteristics and pathogenic potential. Scientific reports 11:6756.
2. Jost BH, Trinh HT, Songer JG. 2006. Clonal relationships among *Clostridium perfringens* of porcine origin as determined by multilocus sequence typing. Veterinary Microbiology 116:158-65.
3. Deguchi A, Miyamoto K, Kuwahara T, Miki Y, Kaneko I, Li J, McClane BA, Akimoto S. 2009. Genetic characterization of type A enterotoxigenic *Clostridium perfringen*s strains. PLoS One 4:e5598.
4. Hibberd MC, Neumann AP, Rehberger TG, Siragusa GR. 2011. Multilocus Sequence Typing Subtypes of Poultry *Clostridium perfringens* Isolates Demonstrate Disease Niche Partitioning. Journal of Clinical Microbiology 49:1556-1567.
